# Developing and validating a lactate-to-albumin ratio-enhanced score for mortality prediction in ICU patients with acute pulmonary embolism: a multi-cohort study

**DOI:** 10.3389/fmed.2026.1819790

**Published:** 2026-06-15

**Authors:** Jun An, Peng Liu, Yingqun Ji

**Affiliations:** 1 Department of Pulmonary and Critical Care Medicine, The First Affiliated Hospital of Dalian Medical University, Dalian, China; 2 Department of Cardiology, The First Affiliated Hospital of Dalian Medical University, Dalian, China; 3 Department of Pulmonary and Critical Care Medicine, Shanghai East Hospital, School of Medicine, Tongji University, Shanghai, China

**Keywords:** acute pulmonary embolism, albumin, intensive care unit, lactate, prediction model, prognosis

## Abstract

**Background:**

The simplified Pulmonary Embolism Severity Index (sPESI) has limited predictive accuracy for critically ill patients with acute pulmonary embolism (APE) and multi-organ dysfunction and lacks dynamic pathophysiological indicators. This study evaluated the predictive value of the lactate-to-albumin ratio (LAR) for 28-day mortality in ICU patients with APE and explored its potential to optimize the sPESI score.

**Methods:**

This study retrospectively analyzed 1098 adult patients with APE in the ICU from the Medical Information Mart for Intensive Care-IV (MIMIC-IV) (derivation cohort, *n* = 306), the eICU Collaborative Research Database (eICU-CRD) (first validation cohort, *n* = 555), and an independent external cohort from the First Affiliated Hospital of Dalian Medical University (second validation cohort, *n* = 237). Multivariable Cox regression, restricted cubic spline (RCS), and Kaplan–Meier survival analyses were used to assess the association between LAR and ICU 28-day all-cause mortality. The predictive efficacy and clinical utility of LAR and the new scoring system (LAR-sPESI) were evaluated using receiver operating characteristic (ROC) curves, Hosmer–Lemeshow (H–L) tests, and decision curve analysis (DCA).

**Results:**

LAR demonstrated a linear relationship with 28-day mortality. The AUC of LAR for predicting mortality was 0.610 in the derivation cohort, 0.710 in the first validation cohort, and 0.647 in the second validation cohort. The LAR-sPESI score showed modest improvements in predictive performance (derivation cohort AUC = 0.650; first validation cohort AUC = 0.733; second validation cohort AUC = 0.701), with acceptable calibration (H–L test, *P* > 0.05 in all cohorts). DCA suggested trends suggestive of potential clinical utility within 15–50% in the derivation cohort, 1%–80% in the first validation cohort, and 3%–80% in the second validation cohort. LAR exhibited moderate discriminative ability for norepinephrine use in all cohorts. In contrast, its predictive performance for pharmacological thrombolysis and mechanical ventilation was limited, with acceptable calibration for all assessable secondary outcomes (all *P* > 0.05).

**Conclusion:**

LAR may serve as a preliminary exploratory predictor of 28-day mortality in ICU patients with APE. The LAR-sPESI score yielded only modest numerical gains in predictive performance, supporting exploratory risk stratification.

## 1 Introduction

Acute pulmonary embolism (APE) is a potentially fatal cardiovascular disease in clinical practice, second only to acute myocardial infarction and stroke. Epidemiological studies indicate that the global incidence of venous thromboembolism (VTE) is about 1/1,000 to 2/1,000 persons per year, among which approximately one-third present as APE, and the incidence increases exponentially with age (1, 2). The overall mortality rate of hospitalized patients with APE is 5–15%, whereas in critically ill patients with hemodynamic instability, this proportion can exceed 30% (3). Therefore, timely and accurate risk stratification is essential for improving the prognosis of APE and guiding treatment decisions.

The simplified Pulmonary Embolism Severity Index (sPESI) is a widely used prognostic assessment tool recommended by international guidelines (3–5). However, its predictive performance is often limited in patients with APE who have multiple organ dysfunction (6–8), because it relies on relatively static clinical indicators such as age, underlying diseases, and vital signs, and fails to capture evolving organ dysfunction dynamically. Even recently developed nomogram models for APE prognosis have highlighted the limitations of traditional scoring systems in integrating dynamic laboratory and treatment-related indicators (9).

The lactate-to-albumin ratio (LAR), a novel composite index, has recently shown advantages in prognostic assessment of critical illness. Lactate reflects the degree of tissue hypoperfusion and cellular hypoxia (10). In contrast, albumin reflects nutritional status, inflammatory response, and vascular endothelial integrity, and exerts protective effects such as antioxidation and free radical scavenging (11, 12). LAR integrates may “damage attack” (elevated lactate) and “defense reserve” (decreased albumin) as a putative composite indicator, thereby achieving a synergistic effect. In various critical conditions such as sepsis, acute myocardial infarction, and trauma, the predictive efficacy of LAR has been shown to be superior to that of a single indicator (13–16). More importantly, LAR is derived from routine laboratory tests, can be dynamically monitored, and is not directly influenced by subjective judgment or treatment intervention, thus demonstrating good clinical applicability (17). It has demonstrated considerable prognostic value in critically ill patients. It is not only superior to the single lactate index but also identified as a key predictor by machine learning models, providing an objective and reliable tool for early risk stratification (18). A recent large-scale retrospective study based on the MIMIC-IV database further confirmed that LAR is an independent predictor of short-term and long-term all-cause mortality in patients with VTE (19).

However, the value of LAR in predicting the prognosis of ICU patients with APE remains underexplored. Given that patients with APE experience pathological processes such as tissue hypoperfusion caused by right heart failure, activation of the inflammation–coagulation cascade, and endothelial injury—all of which are mechanisms that LAR can reflect—LAR is likely to become a valuable supplementary indicator to compensate for the limitations of sPESI. Therefore, based on two major public databases, the Medical Information Mart for Intensive Care (MIMIC-IV) and the eICU Collaborative Research Database (eICU-CRD), together with an additional independent external cohort from our institution, this study aimed to: (1) systematically evaluate the predictive value of LAR for 28-day mortality; (2) construct a new scoring system integrating LAR (LAR-sPESI); (3) validate the predictive efficacy and clinical applicability of the new model through two independent validation cohorts; and (4) explore the predictive value of LAR for secondary outcomes related to clinical therapeutic interventions in ICU patients with APE, thereby providing new tools and evidence for improved risk stratification of ICU patients with APE.

## 2 Materials and methods

### 2.1 Data sources and study design

This study was a retrospective cohort analysis based on three datasets. The derivation cohort data were obtained from the MIMIC-IV (version 2.2) database, which was jointly established by the Computational Physiology Laboratory of the Massachusetts Institute of Technology, Beth Israel Deaconess Medical Center, and Philips Healthcare. It contains detailed medical records of approximately 250,000 ICU patients at Beth Israel Deaconess Medical Center from 2008 to 2019. The first external validation cohort data were derived from the eICU-CRD (version 2.0), which covers clinical data from more than 200,000 patients across 335 ICUs in 208 hospitals in the United States from 2014 to 2015. Both databases are open-access, de-identified clinical datasets and are widely used in critical care research. Researcher LIU completed the required online training and obtained data usage authorization (Certificate Number: 43105842). The second external validation cohort included 382,278 consecutive ICU admissions recorded at the First Affiliated Hospital of Dalian Medical University (DLMU) from January 2018 to December 2023. The study was approved by the institutional review board of the First Affiliated Hospital of DLMU (Approval Number: PJ-KS-KY-2026-165). The study adhered to the Helsinki Declaration and international ethical standards for retrospective studies. All data usage strictly complied with database access agreements to ensure patient privacy and data security.

### 2.2 Inclusion and exclusion criteria

Patients diagnosed with APE were identified using International Classification of Diseases (ICD) codes: ICD-9 codes 41511, 41512, 41519 and ICD-10 codes I26, I260, I2601, I2609, I269, I2690, I2693, I2694, I2699 in MIMIC-IV; and specific diagnosis codes (2768, 2771, 2772, 2773, 2774, 2775, 2776, 2777, 2778, 2779, 2780, 2781) in the eICU database, which are eICU-specific identifiers for the corresponding condition as defined in the eICU data dictionary.

In the DLMU database, patients diagnosed with APE were identified using ICD-10 codes (I26, I26.0, I26.9, I26.900x001, I26.900x002, I26.900x003, I26.900x005, I26.900x006, I26.900x007, I26.900x009, I26.900x010, I26.900x011, I26.900x012, I26.900x013, I26.900x015, I26.901), which represent the Chinese national standardized coding system with additional granularity for clinical specificity. To ensure diagnostic accuracy, all cases were further validated by imaging confirmation (computed tomography pulmonary angiography or ventilation/perfusion scans) in accordance with the 2025 Chinese guidelines for the diagnosis and management of pulmonary embolism (4). Although coding systems varied slightly across databases, all approaches are validated methods for identifying APE in large-scale studies, and the consistency of baseline characteristics across cohorts (Table 1) supports the comparability of case identification.

The inclusion criteria were: (1) adult patients (age ≥ 18 years) diagnosed with APE; (2) first ICU admission during hospitalization with a hospital stay ≥ 24 h; and (3) availability of serum lactate and albumin measurements within 24 h of ICU admission. The exclusion criteria were: (1) multiple ICU admissions during the same hospitalization (only data from the first ICU admission were retained); (2) missing > 20% of core variables required for calculating the sPESI score (age, heart rate, systolic blood pressure, oxygen saturation (SpO_2_), history of chronic cardiopulmonary disease, and malignant tumor); and (3) lack of 28-day follow-up outcome data.

### 2.3 Data extraction and variable definition

Structured Query Language (SQL) was used to extract patient information from the MIMIC-IV and eICU databases. The extracted variables included:

(1) Demographic characteristics: age, sex, race, and body mass index (BMI).
(2) Vital signs: the first measurement and maximum/minimum values of heart rate, respiratory rate, systolic blood pressure, diastolic blood pressure, mean arterial pressure, body temperature, and SpO_2_ within 24 h of ICU admission.
(3) Comorbidities: chronic heart failure, chronic obstructive pulmonary disease, previous myocardial infarction, peripheral vascular disease, diabetes, malignancy, kidney disease, and others.
(4) Laboratory indicators: the first measurement and maximum/minimum values of serum lactate (mmol/L), albumin (g/dL), white blood cell count, red blood cell count, platelet count, blood urea nitrogen (BUN), serum creatinine, blood glucose, international normalized ratio (INR), D-dimer, and arterial blood gas parameters (pH, partial pressure of carbon dioxide (PaCO_2_), partial pressure of oxygen (PaO_2_) and base excess) within 24 h of ICU admission.
(5) Disease severity scores: simplified Pulmonary Embolism Severity Index (sPESI), Simplified Acute Physiology Score II (SAPS II), and Glasgow Coma Scale (GCS) score (defined as the lowest value recorded within 24 h of ICU admission).
(6) Treatment measures: mechanical ventilation, norepinephrine use, and pharmacological thrombolysis.

The primary outcome was uniformly defined across all three cohorts as 28-day all-cause mortality after ICU admission in patients with APE, serving as the key prognostic endpoint for risk stratification in critically ill populations. Secondary outcomes included three clinically relevant therapeutic intervention endpoints during the index ICU stay: norepinephrine use (vasoactive support), receipt of pharmacological thrombolysis, and requirement for mechanical ventilation (invasive or non-invasive), which were selected to reflect clinical severity and supportive care needs and to complement the primary mortality endpoint. The LAR was consistently defined across all cohorts as the ratio of the first measured serum lactate concentration (mmol/L) to the first measured serum albumin concentration (g/dL) within 24 h after ICU admission.

In the DLMU database, patient demographics (age and sex) were extracted from electronic admission records. All additional baseline characteristics within the first 24 h of ICU admission were collected using identical standardized definitions across the MIMIC-IV, eICU-CRD, and DLMU cohorts, including vital signs, core laboratory parameters, and disease severity scores. Unified definitions and acquisition timeframes for key study variables across the three cohorts are provided in Supplementary Table 1.

### 2.4 Statistical analysis

All statistical analyses were performed using R software (version 4.2.3) and SPSS software (version 26.0). A two-sided *P*-value < 0.05 was considered statistically significant.

#### 2.4.1 Comparison of baseline characteristics

The Shapiro–Wilk test was used to assess the normality of continuous variables. Normally distributed variables were presented as mean ± standard deviation and compared using the independent samples *t*-test, whereas non-normally distributed variables were presented as median (interquartile range, IQR) and compared using the Mann–Whitney U test. Categorical variables were expressed as frequency (percentage) and compared using the Chi-square test or Fisher’s exact test.

#### 2.4.2 Association between LAR and mortality

Univariate Cox regression was used to identify potential risk factors. Multivariable Cox regression analysis was applied to determine independent risk factors for 28-day mortality (*P* < 0.05). Restricted cubic splines (RCS) were used to evaluate the nonlinear relationship between LAR and 28-day mortality.

#### 2.4.3 Survival analysis

For exploratory survival analysis, patients were divided into low, moderate, and high LAR groups based on the 25th and 75th percentiles. Kaplan–Meier curves were generated to analyze the association between LAR groups and mortality, and differences were compared using the log-rank test.

#### 2.4.4 Predictive model construction

In the derivation cohort (MIMIC-IV), LAR was incorporated as a continuous variable and combined with the sPESI score to construct the combined prognostic model (LAR-sPESI). This approach preserved the full quantitative information of LAR without dichotomization or cutoff selection. The model was internally validated using bootstrapping and externally validated in the two validation cohorts.

#### 2.4.5 Model performance evaluation

Discrimination: The area under the receiver operating characteristic (ROC) curve (AUC) was calculated for LAR, sPESI, and LAR-sPESI in predicting 28-day mortality in all cohorts.

Calibration: The Hosmer–Lemeshow (H–L) test was used to assess model calibration, and calibration curves were plotted to visually assess the agreement between predicted and observed probabilities. Bootstrap resampling was performed to obtain bias-corrected calibration estimates.

Clinical utility: Decision curve analysis (DCA) was used to evaluate the clinical net benefit of different models across various risk thresholds, and clinical impact curves were plotted.

The above evaluation methods (ROC curves and H–L tests) were also applied to assess the discriminative ability and calibration of LAR for predicting prespecified secondary outcomes across all cohorts.

Patients with complete data on all key indicators required for model construction (including LAR, sPESI) and covariates for multivariable Cox regression adjustment were included in the analyses.

## 3 Results

### 3.1 Baseline characteristics

**FIGURE 1:**
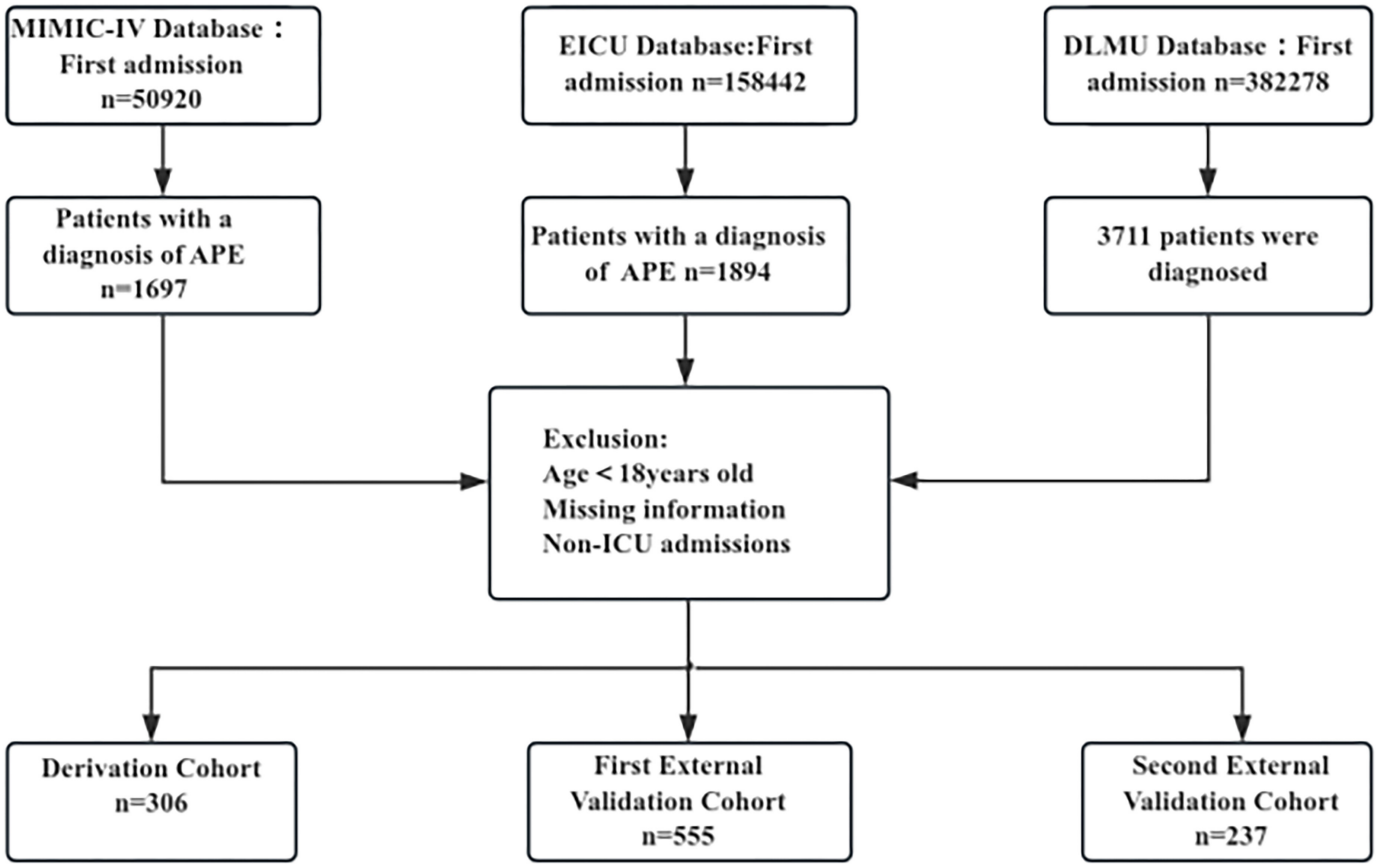
Flowchart of study participants.

This study included a total of 1098 ICU patients with APE, among which 306 cases (27.87%) were in the derivation cohort, 555 (50.55%) in the first validation cohort, and 237 (21.58%) in the second validation cohort (Figure 1). The baseline characteristics of the derivation and first validation cohorts are summarized in Table 1A. Compared with the first validation cohort, patients in the derivation cohort had higher SAPS II scores and GCS scores, as well as a higher prevalence of comorbidities such as myocardial infarction, heart failure, and chronic pulmonary disease (all *P* < 0.05).

**TABLE 1A:** Baseline characteristics of patients in the derivation (MIMIC-IV) and first validation (eICU-CRD) cohorts.

Variables	Total (*n* = 861)	Derivation cohort (*n* = 306)	First external validation cohort (*n* = 555)	*P*
Demographic characteristics				
Age (years)	62.91 ± 16.52	62.66 ± 17.29	63.05 ± 16.10	0.740
Male, n (%)	449 (52.15)	161 (52.61)	288 (51.89)	0.839
Vital signs				
Heart Rate (beats/min)	102.00 (87.00, 117.00)	101.00 (84.00, 118.00)	102.00 (89.00, 116.00)	0.731
SBP (mmHg)	117.00 (98.00, 135.00)	120.00 (103.00, 138.00)	105.50 (83.75, 128.25)	< **0.001**
SpO_2_(%)	95.14 ± 7.77	94.88 ± 10.00	95.29 ± 6.19	0.454
Comorbidities (n%)				
Myocardial infarct	67 (7.78)	37 (12.09)	30 (5.41)	< **0.001**
Congestive heart failure	167 (19.40)	91 (29.74)	76 (13.69)	< **0.001**
Peripheral vascular disease	42 (4.88)	26 (8.50)	16 (2.88)	< **0.001**
Chronic pulmonary disease	106 (12.31)	88 (28.76)	18 (3.24)	< **0.001**
Diabetes	220 (25.55)	79 (25.82)	141 (25.41)	0.895
AKI	370 (42.97)	51 (16.67)	319 (57.48)	< **0.001**
Cancer	193 (22.42)	80 (26.14)	113 (20.36)	0.051
Clinical severity scores				
SAPS II	37.00 (27.00, 52.00)	42.00 (32.00, 56.00)	34.00 (25.00, 48.00)	< **0.001**
GCS	15.00 (11.00, 15.00)	15.00 (15.00, 15.00)	14.00 (6.00, 15.00)	< **0.001**
sPESI	1.00 (1.00, 2.00)	1.00 (1.00, 2.00)	1.00 (0.00, 2.00)	**0.002**
Laboratory parameters				
Lactate (mmol/L)	2.20 (1.30, 4.00)	2.10 (1.30, 4.10)	2.20 (1.20, 4.00)	0.709
Albumin (g/dL)	2.80 (2.40, 3.30)	2.80 (2.40, 3.20)	2.90 (2.35, 3.30)	0.519
LAR	0.786(0.453, 1.520)	0.792 (0.469, 1.495)	0.778 (0.443, 1.531)	0.639
Base excess (mmol/L)	0.00 (−3.37, 2.62)	0.00 (−4.00, 2.00)	0.80 (−2.77, 3.20)	< **0.001**
PaCO_2_ (mmHg)	43.00 (36.00, 53.00)	44.00 (38.00, 54.00)	41.00 (35.00, 51.40)	**0.004**
PaO_2_ (mmHg)	116.10 (77.00, 194.68)	109.00 (68.25, 186.75)	124.50 (83.38, 199.00)	**0.001**
pH	7.42 (7.36, 7.46)	7.40 (7.34, 7.45)	7.43 (7.38, 7.47)	< **0.001**
WBC, × 10^9^/L	13.30 (9.20, 18.30)	14.20 (10.22, 19.67)	12.50 (8.80, 17.30)	< **0.001**
RBC, × 10^9^/L	3.94 (3.31, 4.51)	3.76 (3.26, 4.30)	4.04 (3.35, 4.63)	< **0.001**
BUN (mg/dL)	25.00 (16.00, 41.00)	23.00 (14.00, 37.00)	26.00 (17.00, 43.00)	**0.021**
Creatinine (mg/dL)	1.20 (0.86, 1.96)	1.10 (0.80, 1.80)	1.27 (0.90, 2.00)	< **0.001**
INR	1.40 (1.20, 1.80)	1.40 (1.20, 1.80)	1.40 (1.20, 1.92)	0.264
Potassium (mmol/L)	4.40 (4.10, 4.90)	4.10 (3.68, 4.80)	4.50 (4.10, 4.90)	**< 0.001**
Secondary outcomes (n%)				
Norepinephrine	445 (51.68)	161 (52.61)	284 (51.17)	0.685
Pharmacological thrombolysis	109 (12.66)	2 (0.65)	107 (19.28)	< **0.001**
Mechanical ventilation	419 (48.66)	270 (88.24)	149 (26.85)	< **0.001**

Data are presented as mean ± SD for normally distributed continuous variables, median (IQR) for non-normally distributed continuous variables, and n (%) for categorical variables. Bold *P* values indicate statistically significant differences (*P* < 0.05). SBP, systolic blood pressure; SpO_2_, oxygen saturation; AKI, acute kidney injury; SAPS II, Simplified Acute Physiology Score II; GCS, Glasgow Coma Scale; sPESI, Simplified Pulmonary Embolism Severity Index; LAR, lactate-to-albumin ratio; PaCO_2_, arterial partial pressure of carbon dioxide; PaO_2_, arterial partial pressure of oxygen; WBC, white blood cell; RBC, red blood cell; BUN, blood urea nitrogen; INR, international normalized ratio.

Table 1B presents the baseline characteristics of the second validation cohort, stratified by LAR based on the 25th and 75th percentiles: Q1 (low, LAR ≤ 0.335, *n* = 60), Q2 (moderate, 0.335 < LAR < 1.002, *n* = 124), and Q3 (high, LAR ≥ 1.002, *n* = 53). The high LAR group had significantly higher SAPS II scores, lower GCS scores, higher rates of mechanical ventilation, and a lower proportion of patients receiving rivaroxaban compared with the low and moderate LAR groups (all *P* < 0.05).

**TABLE 1B:** Baseline characteristics of patients in the second validation (DLMU) cohort.

Variables	Total (*n* = 237)	Q1 (*n* = 60)	Q2 (*n* = 124)	Q3 (*n* = 53)	*P*
Demographic characteristics					
Age (years)	70.71 ± 13.14	70.88 ± 11.45	70.01 ± 13.51	72.15 ± 14.13	0.608
Male, n (%)	130 (54.85)	34 (56.67)	67 (54.03)	29 (54.72)	0.945
Vital signs					
Heart Rate (beats/min)	90.0(78.0, 105.0)	85.0(71.3 104.5)	91.0 (80.0, 105.8)	90 (80.0, 106.5)	0.298
SBP (mmHg)	131.11 ± 28.00	135.42 ± 30.35	133.88 ± 25.83	119.65 ± 27.40	**0.004**
SpO_2_(%)	87.74 ± 19.57	86.13 ± 20.61	89.30 ± 16.42	85.87 ± 24.52	0.446
Comorbidities (n%)					
Myocardial infarct	59 (24.89)	13 (21.67)	28 (22.58)	18 (33.96)	0.221
Congestive heart failure	61 (25.74)	13 (21.67)	30 (24.19)	18 (33.96)	0.279
Peripheral vascular disease	150 (63.29)	40 (66.67)	79 (63.71)	31 (58.49)	0.661
Chronic pulmonary disease	31 (13.08)	13 (21.67)	15 (12.10)	3 (5.66)	**0.038**
Diabetes	32 (13.50)	7 (11.67)	20 (16.13)	5 (9.43)	0.437
AKI	40 (16.88)	9 (15.00)	18 (14.52)	13 (24.53)	0.240
Cancer	46 (19.41)	16 (26.67)	22 (17.74)	8 (15.09)	0.238
Clinical severity scores					
SAPS II	37.00 (27.00, 51.00)	33.50 (24.00,48.25)	37.00 (27.00,48.00)	45.00 (33.00,58.00)	**0.002**
GCS	15.00 (8.00, 15.00)	15.00 (10.25,15.00)	15.00 (10.00,15.00)	10.00 (5.00,15.00)	**0.002**
sPESI, n(%)					0.088
0	62 (26.16)	20 (33.33)	35 (28.23)	7 (13.21)	
1	66 (27.85)	17 (28.33)	36 (29.03)	13 (24.53)	
2	65 (27.43)	15 (25.00)	33 (26.61)	17 (32.08)	
3	32 (13.50)	5 (8.33)	17 (13.71)	10 (18.87)	
4	12 (5.06)	3 (5.00)	3 (2.42)	6 (11.32)	
Laboratory parameters					
Albumin (g/dL)	3.40 (2.91, 3.81)	3.49 (3.25, 3.88)	3.40 (2.94, 3.77)	3.05 (2.52, 3.81)	**0.007**
PaCO_2_ (mmHg)	36.19 ± 10.93	38.14 ± 10.44	36.79 ± 11.39	32.55 ± 9.67	**0.020**
PaO_2_ (mmHg)	103.37 ± 49.40	100.19 ± 31.86	102.93 ± 50.10	108.01 ± 63.01	0.711
WBC, × 10^9^/L	10.17 (6.70, 14.08)	11.19 (8.22,16.53)	9.45 (6.18,12.80)	10.04 (6.14,14.44)	**0.020**
RBC, × 10^9^/L	3.92 (3.36, 4.46)	3.97 (3.44,4.35)	4.01 (3.43,4.50)	3.69 (2.84,4.19)	0.112
Urea (mmol/L)	7.05 (5.13, 11.63)	7.38 (5.21,10.09)	6.79 (4.81,12.56)	7.69 (5.67,14.11)	0.210
Creatinine (μmol/L)	76.00 (59.00, 101.00)	70.00 (56.00,90.00)	73.50 (56.00,92.25)	88.00 (69.00,144.00)	**0.006**
INR	1.10 (1.00, 1.26)	1.08 (1.01,1.23)	1.08 (1.00,1.23)	1.16 (1.06,1.31)	**0.049**
Potassium (mmol/L)	3.90 (3.53, 4.25)	3.90 (3.50,4.16)	3.90 (3.52,4.25)	3.90 (3.60,4.36)	0.581
Antithrombotic agents (n%)					
Heparin	198 (83.54)	53 (88.33)	103 (83.06)	42 (79.25)	0.420
Warfarin	18 (7.59)	5 (8.33)	12 (9.68)	1 (1.89)	0.189
Rivaroxaban	85 (36.48)	31 (51.67)	44 (36.67)	10 (18.87)	**0.001**
Secondary outcomes (n%)					
Norepinephrine	84 (35.44)	24 (40.00)	41 (33.06)	19 (35.85)	0.652
Pharmacological thrombolysis	35 (14.77)	8 (13.33)	20 (16.13)	7 (13.21)	0.826
Mechanical ventilation	115 (48.52)	19 (31.67)	58 (46.77)	38 (71.70)	**< 0.001**

Data are presented as mean ± SD for normally distributed continuous variables, median (IQR) for non-normally distributed continuous variables, and n (%) for categorical variables. Q 1: LAR ≤ 0.335; Q 2: 0.335 < LAR < 1.002; Q 3: LAR ≥ 1.002. BUN (mg/dL) = Urea (mmol/L) × 2.8. Creatinine (mg/dL) = creatinine (μmol/L)/88.4. Bold *P* values indicate statistically significant differences (*P* < 0.05). SBP, systolic blood pressure; SpO2, oxygen saturation; AKI, acute kidney injury; SAPS II, Simplified Acute Physiology Score II; GCS, Glasgow Coma Scale; sPESI, Simplified Pulmonary Embolism Severity Index; LAR, lactate-to-albumin ratio; PaCO_2_, arterial partial pressure of carbon dioxide; PaO2, arterial partial pressure of oxygen; WBC, white blood cell; RBC, red blood cell; BUN, blood urea nitrogen; INR, international normalized ratio.

### 3.2 Relationship between LAR and 28-day mortality

**FIGURE 2:**
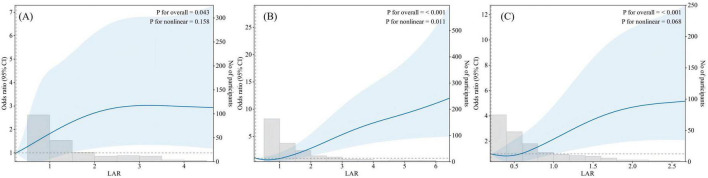
RCS analysis curves. RCS analysis curves for the derivation cohort **(A)**, first validation cohort **(B)**, and second validation cohort **(C)**.

RCS analysis demonstrated a linear association between LAR and 28-day mortality in the derivation cohort (overall *P* = 0.043, *P* for nonlinearity = 0.158). In the first validation cohort, RCS analysis identified a highly significant association (overall *P* < 0.001) with a significant nonlinear trend (*P* for nonlinearity = 0.011), whereas a linear association was observed again in the second validation cohort (overall *P* < 0.001, *P* for nonlinearity = 0.068). Across all three cohorts, the risk of 28-day mortality increased progressively with increasing LAR values (Figures 2A–C). For exploratory survival analysis, patients were stratified into three LAR groups based on the cohort-specific 25th and 75th percentiles in each cohort separately. In the derivation cohort: Q1 (low, LAR ≤ 0.469, *n* = 77), Q2 (moderate, 0.469 < LAR ≤ 1.495, *n* = 152), and Q3 (high, LAR > 1.495, *n* = 77). In the first validation cohort: Q1 (low, LAR ≤ 0.443, *n* = 139), Q2 (moderate, 0.443 < LAR ≤ 1.531, *n* = 277), and Q3 (high, LAR > 1.531, *n* = 139). In the second validation cohort: Q1 (low, LAR ≤ 0.335, *n* = 60), Q2 (moderate, 0.335 < LAR ≤ 1.002, *n* = 124), and Q3 (high, LAR > 1.002, *n* = 53).

The results of multivariable Cox regression analysis for 28-day mortality are presented in Table 2. All hazard ratios (HRs) for Q1 and Q2 were less than 1 relative to Q3 across all models and cohorts. In the derivation cohort, Q1 and Q2 showed significantly lower mortality risk in unadjusted and partially adjusted models, whereas the fully adjusted model (Model 3) did not reach statistical significance. In the first validation cohort, Q1 and Q2 remained associated with significantly lower mortality risk in fully adjusted models (all *P* < 0.001). In the second validation cohort, findings from fully adjusted models were consistent with those from Cox regression, showing lower mortality risk in Q1 and Q2 (all P ≤ 0.002).

**TABLE 2:** Cox proportional hazards regression analysis for 28-day mortality period in patients with acute pulmonary embolism.

Variables	N	Model 1 (Unadjusted)		Model 2 (Partially adjusted)		Model 3 (Fully adjusted)	
		HR (95% CI)	P	HR (95% CI)	P	HR (95% CI)	P
Derivation cohort							
LAR Q1	63	0.455 (0.241–0.860)	0.015	0.520 (0.274–0.988)	0.046	0.574 (0.300–1.097)	0.093
LAR Q2	153	0.595 (0.377–0.939)	0.026	0.659 (0.415–1.044)	0.076	0.702 (0.441–1.118)	0.136
LAR Q3	90	Reference	–	Reference	–	Reference	–
First validation cohort							
LAR Q1	139	0.193 (0.112–0.335)	< 0.001	0.189 (0.109–0.328)	< 0.001	0.368 (0.206–0.657)	< 0.001
LAR Q2	277	0.217 (0.143–0.328)	< 0.001	0.209 (0.138–0.318)	< 0.001	0.334 (0.215–0.517)	< 0.001
LAR Q3	139	Reference	–	Reference	–	Reference	–
Second validation cohort							
LAR Q1	60	0.298 (0.135–0.638)	0.002	0.290 (0.130–0.626)	0.002	0.284 (0.126–0.616)	0.002
LAR Q2	124	0.293 (0.147–0.569)	< 0.001	0.291 (0.144–0.571)	< 0.001	0.286 (0.141–0.564)	< 0.001
LAR Q3	53	Reference	–	Reference	–	Reference	–

Model 1: Unadjusted. Model 2: Adjusted for sex, age, and diabetes mellitus. Model 3: Additionally adjusted for congestive heart failure, malignancy, mechanical ventilation, and acute kidney injury. LAR Q3 served as the reference group. CI, confidence interval; HR, hazard ratio; LAR, lactate-to-albumin ratio; OR, odds ratio.

**FIGURE 3:**
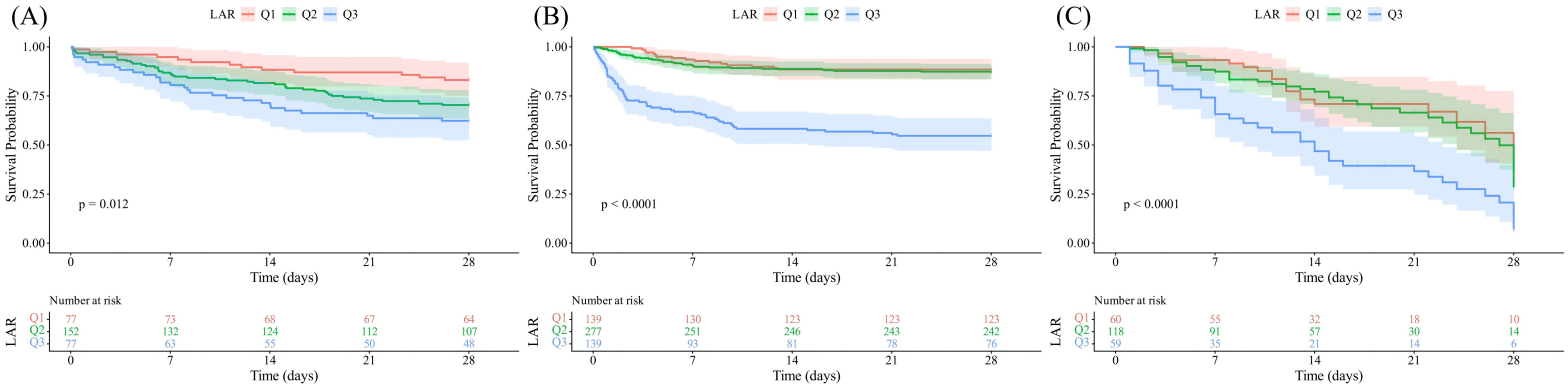
Kaplan–Meier survival curves. Kaplan–Meier survival curves for 28-day mortality by LAR groups (based on the 25th and 75th percentiles) in the derivation cohort **(A)**, first validation cohort **(B)**, and second validation cohort **(C)**. **(A)** Derivation cohort: Q1 ≤ 0.469, Q2 0.469–1.495, Q3 > 1.495; log-rank *P* = 0.012. **(B)** First validation cohort: Q1 ≤ 0.443, Q2 0.443–1.531, Q3 > 1.531; log-rank *P* < 0.0001. **(C)** Second validation cohort: Q1 ≤ 0.335, Q2 0.335–1.002, Q3 > 1.002; log-rank *P* < 0.0001.

Kaplan–Meier survival analysis showed significantly higher 28-day cumulative mortality in the high LAR group across all cohorts (log-rank *P* = 0.012 for the derivation cohort, *P* < 0.0001 for the first validation cohort, and *P* < 0.0001 for the second validation cohort; Figures 3A–C).

### 3.3 Predictive performance of different scores

The discriminative ability (AUC) of different scoring systems for predicting 28-day mortality was compared. In the derivation cohort, the AUC for the sPESI score was 0.618 (95% confidence interval (CI): 0.551–0.686), for albumin was 0.563 (95% CI: 0.494–0.632), for lactate was 0.605 (95% CI: 0.531–0.679), and for LAR was 0.610 (95% CI: 0.540–0.680). In the first validation cohort, predictive performance was more favorable. The AUC for sPESI was 0.593 (95% CI: 0.538–0.648), for albumin was 0.621 (95% CI: 0.564–0.678), for lactate was 0.686 (95% CI: 0.622–0.749), and for LAR was 0.710 (95% CI: 0.650–0.771).

**FIGURE 4:**
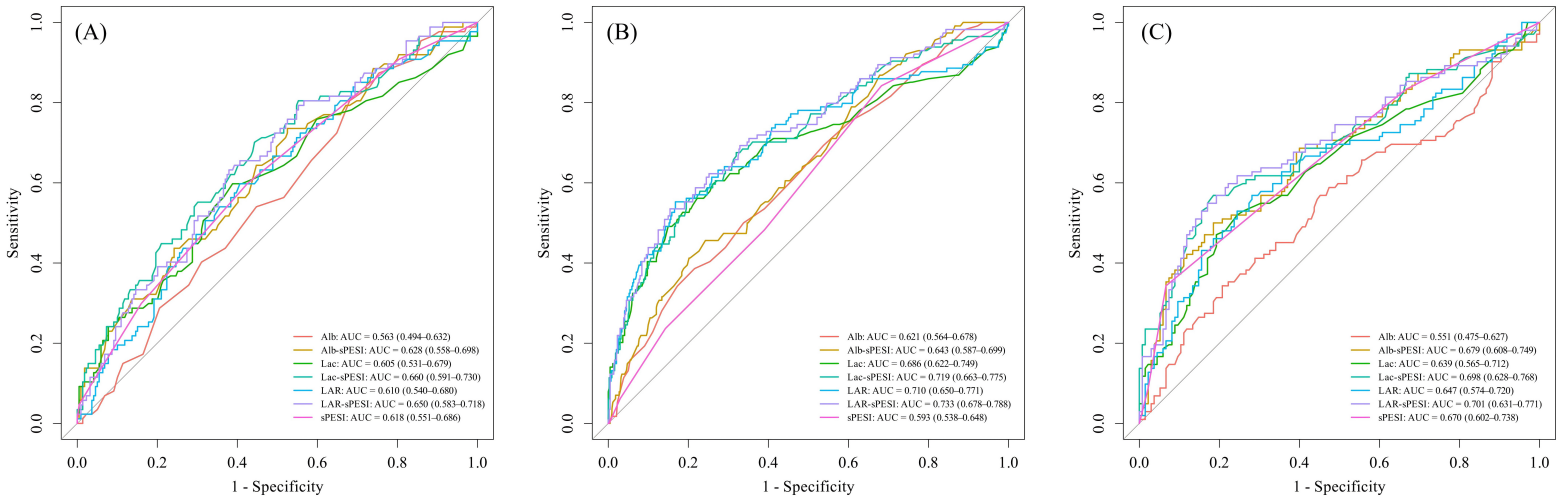
ROC curves of different scoring systems for predicting 28-day mortality in the derivation cohort **(A)**, first validation cohort **(B)**, and second validation cohort **(C)**. AUC values are presented with 95% confidence intervals. Alb, albumin; Lac, lactate; LAR, lactate-to-albumin ratio; Alb-sPESI, albumin combined with sPESI; Lac-sPESI, lactate combined with sPESI; LAR-sPESI, lactate-to-albumin ratio combined with sPESI.

The discriminative ability of these predictors was further evaluated in the second independent validation cohort. The AUC values for albumin, sPESI, lactate, and LAR were 0.551, 0.670, 0.639, and 0.647, respectively. The LAR-sPESI score, which integrates LAR into sPESI, showed a slightly higher AUC of 0.701 in this cohort. The performance of the LAR-sPESI model was consistent across all three cohorts: derivation cohort AUC = 0.650 (95% CI: 0.583–0.718) and first validation cohort AUC = 0.733 (95% CI: 0.678–0.788) (Figures 4A–C).

### 3.4 Construction and validation of the new scoring system (LAR-sPESI)

LAR was incorporated as a continuous variable and combined with the sPESI score to construct the LAR-sPESI prognostic model. This model retained the full quantitative information of LAR and was internally validated in the derivation cohort and externally validated in the two validation cohorts.

**FIGURE 5:**
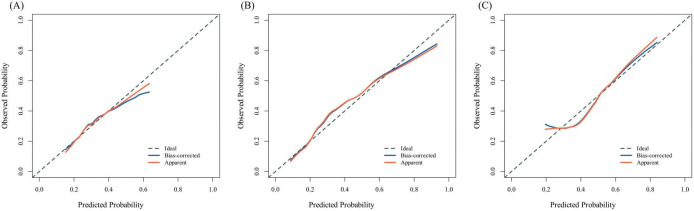
Calibration plots of the LAR-sPESI model for predicting 28-day mortality in the derivation cohort **(A)**, first validation cohort **(B)**, and second validation cohort **(C)**. The Hosmer–Lemeshow test was non-significant in all cohorts (*P* > 0.05).

**FIGURE 6:**
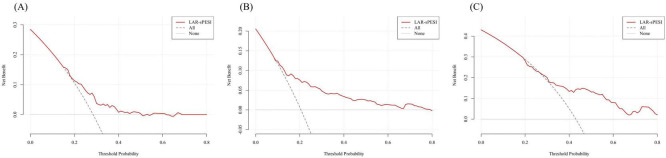
Decision curve analysis of the LAR-sPESI model for predicting 28-day mortality in the derivation cohort **(A)**, first validation cohort **(B)**, and second validation cohort **(C)**.

The AUC of LAR-sPESI increased to 0.650 (95% CI: 0.583–0.718) in the derivation cohort (MIMIC-IV), 0.733 (95% CI: 0.678–0.788) in the first validation cohort (eICU-CRD), and 0.701 (95% CI: 0.631–0.771) in the second validation cohort (DLMU-ICU cohort), with numerical increases of 0.032, 0.14, and 0.031 compared with the original sPESI, respectively (Figures 4A–C). The H–L test yielded non-significant results in all cohorts (derivation: χ^2^ = 8.292, df = 8, *P* = 0.405; first validation: χ^2^ = 6.489, df = 8, *P* = 0.593; second validation: χ^2^ = 11.872, df = 8, *P* = 0.157). Calibration curves were closely aligned with the ideal diagonal across all cohorts, indicating no evidence of lack of fit (Figures 5A–C). Decision curve analysis suggested trends suggestive of potential clinical utility for the LAR-sPESI model within 15–50% in the derivation cohort, 10%–80% in the first validation cohort, and 30–80% in the second validation cohort (Figures 6A–C).

### 3.5 Secondary outcomes predictive performance

**FIGURE 7:**
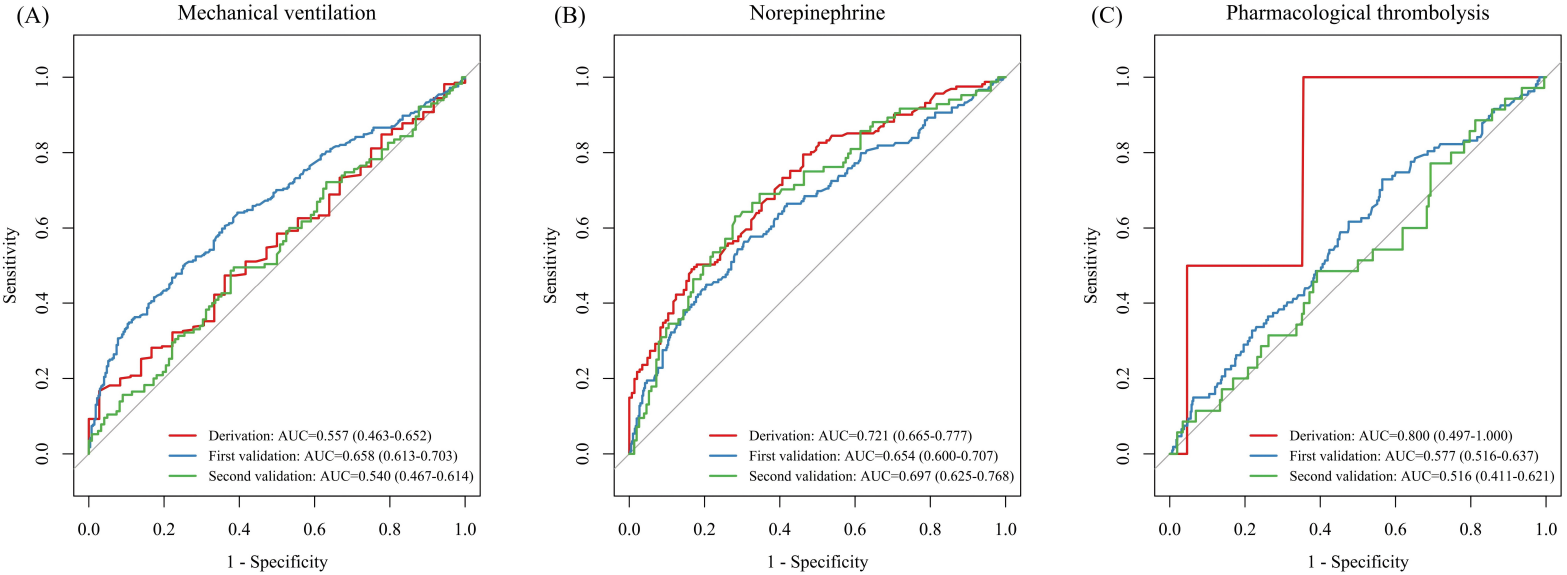
ROC curves of LAR for predicting the three predefined secondary outcomes during ICU stay: mechanical ventilation **(A)**, norepinephrine use **(B)**, and pharmacological thrombolysis **(C)**. Each panel includes the derivation, first validation, and second validation cohorts.

The baseline incidence of the three secondary outcomes (norepinephrine use, pharmacological thrombolysis, and mechanical ventilation) across all cohorts is presented in Tables 1A,B, and the discriminative ability of LAR for these outcomes was further analyzed. The predictive ability of LAR for three prespecified secondary outcomes (norepinephrine use, receipt of pharmacological thrombolysis, and requirement for mechanical ventilation) was evaluated across the derivation, first validation, and second validation cohorts (Figures 7A–C). LAR showed moderate discriminative ability for norepinephrine use, with AUC values of 0.721 (95% CI: 0.665–0.777) in the derivation cohort, 0.654 (95% CI: 0.600–0.707) in the first validation cohort, and 0.697 (95% CI: 0.625–0.768) in the second validation cohort. For pharmacological thrombolysis and mechanical ventilation, the discriminative performance of LAR was variable, with corresponding AUC ranges of 0.516–0.800 and 0.540–0.658, respectively. The H–L test confirmed satisfactory calibration for all assessable secondary outcomes (all *P* > 0.05), with consistent degrees of freedom (df = 8) for norepinephrine use and mechanical ventilation across cohorts. For pharmacological thrombolysis, df = 5 was applied in the second validation cohort; calibration curve analysis was not feasible in the derivation cohort because of an extremely low event count (*n* = 2).

## 4 Discussion

### 4.1 Main findings and innovative significance

Based on 1098 ICU patients with APE from the MIMIC-IV, eICU, and an independent hospital database, this study systematically integrated LAR into the sPESI system, constructed the LAR-sPESI model, and achieved three modest numerical advancements. First, a linear relationship between LAR and 28-day mortality was observed, indicating that higher LAR values were associated with an increased mortality risk. Second, the LAR-sPESI score increased its AUC from 0.618 to 0.650 (Δ = 0.032) in the derivation cohort, representing a modest improvement in discrimination. Third, this modest increase in predictive performance was consistently observed in two external validation cohorts, including a single-center cohort from our institution. Kaplan–Meier curve analysis across all cohorts indicated that higher LAR levels corresponded to progressively lower 28-day survival (log-rank *P* = 0.012 for the derivation cohort, *P* < 0.0001 for the first and second validation cohorts), numerically supporting the potential of the model for risk stratification. This independent validation helps to alleviate concerns of overfitting and suggests that the model may be generalizable to broader clinical settings. LAR also showed moderate discriminative ability for norepinephrine use and limited performance for other secondary outcomes, indicating its role as an auxiliary biomarker for early identification of ICU patients with APE requiring vasoactive support.

Notably, the fully adjusted model in the derivation cohort was not statistically significant. In contrast, both validation cohorts showed consistently significant results, providing exploratory, hypothesis-generating support for the value of LAR in risk stratification.

### 4.2 Mechanistic interpretation of the association between LAR and mortality

This study suggested a significant dose–response relationship between LAR and 28-day mortality across all cohorts. The risk of death was consistently highest in the high LAR group, whereas the risk gradually decreased in the moderate and low LAR groups. This finding appears consistent with the established understanding of critical care pathophysiology. Elevated blood lactate is a marker of tissue hypoperfusion and cellular hypoxia. A recent meta-analysis by Wang et al. demonstrated that elevated lactate levels were associated with a 5.13-fold increased risk of all-cause mortality in unselected patients with APE and a 4.54-fold increased risk even in hemodynamically stable patients (20). Previous studies (21–23) have shown that lactate can exacerbate prothrombotic states and the inflammation–coagulation cycle by promoting neutrophil extracellular trap formation, enhancing thrombin activity, and delaying fibrinolysis.

As a composite index, the core value of LAR lies in simultaneously reflecting the intensity of metabolic stress and the reserve of nutritional defense. Its mechanisms may include the following three aspects. First, high lactate directly indicates insufficient tissue perfusion and cellular energy deficit. Second, low albumin is not only a marker of systemic inflammation but also reflects deterioration of nutritional status and impairment of hepatic synthetic function (24–27). During systemic inflammatory responses, albumin synthesis is inhibited, extravascular leakage increases, and the combined effects of increased catabolism lead to a marked reduction in its level, reflecting depletion of nutritional reserves and ongoing protein catabolism (28). Moreover, albumin possesses direct antithrombotic properties, including inhibition of platelet aggregation and enhancement of antithrombin III activity, and hypoalbuminemia has been independently associated with increased mortality risk in patients with APE (29). Third, a high LAR may reflect a severe imbalance in the “injury–defense” system, indicating that patients are simultaneously exposed to increased metabolic stress and depletion of nutritional reserves.

This comprehensive quantification of core pathophysiological processes—tissue perfusion, inflammation, endothelial integrity, and nutritional reserve—may help address the inherent limitations of sPESI in the ICU setting. The sPESI score relies on relatively static clinical indicators such as age, comorbidities, and vital signs, which fail to capture the dynamic progression of organ dysfunction and metabolic derangement in critically ill patients with APE (6–8). In contrast, LAR integrates two routinely measured laboratory parameters that reflect ongoing tissue hypoperfusion and systemic inflammatory burden, serving as a dynamic physiological marker of the “injury–defense imbalance.” This putative mechanistic relationship may partly explain why integrating LAR into the sPESI framework yields modest incremental improvements in predictive performance.

The unique value of LAR in ICU patients with APE is further supported by its ability to detect early metabolic decompensation before overt clinical deterioration. A nationwide cohort study of over 230,000 ICU patients demonstrated that LAR showed superior predictive accuracy for in-hospital mortality compared with lactate or albumin alone, with an AUC of 0.761 (30). This finding reinforces the generalizability of LAR as a risk marker across diverse critically ill populations. Notably, the cohort-specific percentile stratification of LAR used for Kaplan–Meier analysis was exploratory and not intended for fixed clinical thresholding. A unified, fixed threshold derived from the derivation cohort will be explored in future studies to enhance reproducibility and standardized external validation.

Additionally, dynamic changes in lactate may provide more comprehensive prognostic information than a single measurement. Ebner et al. demonstrated that venous lactate improved the prediction of in-hospital adverse outcomes in normotensive patients with APE (31). Combined with the present findings, it is suggested that dynamic LAR monitoring may more accurately capture the evolution of the “injury–defense balance” than a single LAR measurement, thereby enabling earlier and more sensitive prognostic assessment.

### 4.3 Improvement in predictive performance with LAR-sPESI

The LAR-sPESI scoring system, developed by incorporating LAR, showed a modest numerical increase in predictive performance. In the derivation cohort, the AUC increased from 0.618 to 0.650 (Δ = 0.032). This improvement was consistently observed in the eICU validation cohort (Δ = 0.14). In the DLMU-ICU cohort, the LAR-sPESI model (AUC = 0.701) again demonstrated better performance compared with the traditional sPESI (AUC = 0.670), confirming the reproducibility of this improvement.

The model showed modest to moderate discriminative ability (AUC 0.650–0.733). For calibration, the H–L tests showed no significant lack of fit across all cohorts (all *P* > 0.05), and calibration curves approximated the ideal diagonal, suggesting overall consistency between predicted and observed probabilities. Decision curve analysis suggested trends suggestive of potential clinical utility within cohort-specific thresholds, supporting its role as a preliminary exploratory adjunctive indicator.

### 4.4 Comparison with previous studies, controversies, and unique contributions

The findings corroborate and extend previous literature in the following aspects:

(1) Composite advantage of LAR: A meta-analysis by Wang et al. confirmed that elevated lactate levels are associated with significantly increased mortality risk in patients with APE, with consistent findings across both unselected and hemodynamically stable populations (20). The present study further suggested that the predictive ability of LAR surpasses that of lactate alone, supporting the notion that it may reflect the dual pathological processes of “tissue perfusion injury” and “nutritional defense depletion.”
(2) Cross-disease applicability: The association between LAR and prognosis has been reported in various critical illnesses such as sepsis and myocardial infarction. Confirmation of this pattern in the APE population suggests that LAR may reflect a common core pathological pathway in critical illness.
(3) Reconfirmation of sPESI limitations: The observed relatively low predictive performance of sPESI in ICU patients with APE is consistent with previous studies (32), underscoring the need for more pathophysiologically relevant predictors in this population.
(4) Innovations of this study: This study is the first to systematically evaluate the prognostic value of LAR in ICU patients with APE and integrate it into the sPESI to construct the LAR-sPESI model. Using a triple-cohort design—including derivation, first external validation (eICU), and a second independent external validation from a distinct clinical setting (DLMU-ICU cohort)—along with multi-dimensional evaluation (RCS, ROC, DCA, H–L test), the model demonstrated modest predictive performance, acceptable calibration, and preliminary exploratory value.

### 4.5 Limitations and future research directions

This study has several limitations. As a retrospective study, it is subject to potential selection bias and confounding factors. Only a single LAR measurement was used, and the impact of dynamic changes on prognosis was not assessed. There is a lack of experimental validation of the underlying mechanisms of LAR, and its clinical translational value has not yet been confirmed through interventional studies. In the derivation cohort, the fully adjusted Cox model did not reach statistical significance, which may be attributed to the relatively small sample size, limited number of events (*n* = 87), and adjustment for multiple covariates, leading to insufficient statistical power. Future research should focus on prospective study designs, exploration of the predictive value of dynamic LAR monitoring, and evaluation through randomized controlled trials of whether LAR-based intervention strategies (such as albumin supplementation or individualized anticoagulation therapy) can improve patient outcomes, thereby supporting further risk stratification research.

## 5 Conclusion

This study confirmed a linear association between LAR and 28-day mortality in ICU patients with APE across the MIMIC-IV and eICU datasets, and an independent external cohort. The LAR-sPESI score showed a modest numerical increase in predictive performance over the traditional sPESI in all cohorts, and LAR-based risk stratification identified significant differences in 28-day survival among patient groups. Additionally, LAR showed moderate predictive ability for norepinephrine use and may help identify ICU patients with APE requiring vasoactive support, with satisfactory calibration for all assessable secondary outcomes. The LAR-sPESI model demonstrated acceptable calibration and trends suggestive of potential clinical utility across multiple independent databases, supporting its role as an exploratory supplementary indicator for early risk assessment in ICU patients with APE.

## Data Availability

The original contributions presented in the study are included in the article/Supplementary material, further inquiries can be directed to the corresponding author.

## References

[1] Wendelboe AM Raskob GE. Global burden of thrombosis: epidemiologic aspects. *Circ Res.* (2016) 118:1340–7. 10.1161/CIRCRESAHA.115.306841 27126645

[2] Zhen K Tao Y Xia L Wang S Gao Q Wang D et al. Epidemiology of pulmonary embolism in China, 2021: a nationwide hospital-based study. *Lancet Reg Health West Pac.* (2025) 54:101258. 10.1016/j.lanwpc.2024.101258 39759425 PMC11699474

[3] Konstantinides SV Meyer G Becattini C Bueno H Geersing GJ Harjola VP et al. 2019 ESC Guidelines for the diagnosis and management of acute pulmonary embolism developed in collaboration with the European Respiratory Society (ERS). *Eur Heart J.* (2019) 41:543–603. 10.1093/eurheartj/ehz405 31504429

[4] Chinese Society of Cardiology, Chinese Medical Association, Editorial Board of Chinese Journal of Cardiology Chinese guidelines for the diagnosis and treatment of acute pulmonary embolism 2025. *Chin J Cardiol.* (2025) 53:587–619. 10.3760/cma.j.cn112148-20250225-00140 40528599

[5] Creager MA Barnes GD Giri J Mukherjee D Jones WS Burnett AE et al. AHA/ACC/ACCP/ACEP/CHEST/SCAI/SHM/SIR/SVM/SVN guideline for the evaluation and management of acute pulmonary embolism in adults: a report of the american college of cardiology/american heart association joint committee on clinical practice guidelines. *Circulation.* (2026) 153:e977–1051. 10.1161/CIR.0000000000001415 41712677

[6] Ryll MJ Zodl A Weingarten TN Rabinstein AA Warner DO Schroeder DR et al. Predicting hospital survival in patients admitted to ICU with pulmonary embolism. *J Intensive Care Med.* (2024) 39:455–64. 10.1177/08850666231212875 37964551 PMC10935623

[7] İdin K Dereli S Kaya A Yenerçağ M Yılmaz AS Tayfur K et al. Modified model for end-stage liver disease score predicts 30-day mortality in high-risk patients with acute pulmonary embolism admitted to intensive care units. *Scand Cardiovasc J.* (2021) 55:237–44. 10.1080/14017431.2021.1876912 33491501

[8] Ergan B Ergün R Çalışkan T Aydın K Tokur ME Savran Y et al. Mortality related risk factors in high-risk pulmonary embolism in the ICU. *Can Respir J.* (2016) 2016:2432808. 10.1155/2016/2432808 28025592 PMC5153485

[9] Ding CW Liu C Zhang ZP Cheng CY Pei GS Jing ZC et al. Development and external validation of a nomogram for predicting short-term prognosis in patients with acute pulmonary embolism. *Int J Cardiol.* (2024) 407:132065. 10.1016/j.ijcard.2024.132065 38642720

[10] Deulkar P Singam A Mudiganti VNKS Jain A. Lactate monitoring in intensive care: a comprehensive review of its utility and interpretation. *Cureus.* (2024) 16:e66356. 10.7759/cureus.66356 39246930 PMC11379417

[11] Wiedermann CJ. Hypoalbuminemia as surrogate and culprit of infections. *Int J Mol Sci.* (2021) 22:4496. 10.3390/ijms22094496 33925831 PMC8123513

[12] Almasaudi AS Dolan RD Edwards CA McMillan DC. Hypoalbuminemia reflects nutritional risk, body composition and systemic inflammation and is independently associated with survival in patients with colorectal cancer. *Cancers.* (2020) 12:1986. 10.3390/cancers12071986 32708140 PMC7409314

[13] Park J Yang WT Yeom SR Park SW Cho YM Tae WU et al. Prognostic value of the lactate-albumin ratio in trauma: a comparison with shock index and injury severity score. *PLoS One.* (2025) 20:e0326367. 10.1371/journal.pone.0326367 40591619 PMC12212560

[14] Lichtenauer M Wernly B Ohnewein B Franz M Kabisch B Muessig J et al. The lactate/albumin ratio: a valuable tool for risk stratification in septic patients admitted to ICU. *Int J Mol Sci.* (2017) 18:1893. 10.3390/ijms18091893 28869492 PMC5618542

[15] Shin J Hwang SY Jo IJ Kim WY Ryoo SM Kang GH et al. Prognostic value of the lactate/albumin ratio for predicting 28-day mortality in critically ILL sepsis patients. *Shock.* (2018) 50:545–50. 10.1097/SHK.0000000000001128 29461463

[16] Mahashabde ML Bhimani YR Bhavsar HM. The correlation between the lactate/albumin ratio and Sequential Organ Failure Assessment (SOFA) score in patients with sepsis and septic shock. *Cureus.* (2024) 16:e65616. 10.7759/cureus.65616 39205773 PMC11357719

[17] Bou Chebl R Geha M Assaf M Kattouf N Haidar S Abdeldaem K et al. The prognostic value of the lactate/albumin ratio for predicting mortality in septic patients presenting to the emergency department: a prospective study. *Ann Med.* (2021) 53:2268–77. 10.1080/07853890.2021.2009125 34854770 PMC8648034

[18] Thanh NT Luan VT. Comparison of serum lactate and lactate-derived ratios as prognostic biomarkers in pediatric dengue shock syndrome using supervised machine learning models. *PLoS One.* (2025) 20:e0335022. 10.1371/journal.pone.0335022 41144436 PMC12558473

[19] Huang Y Zhang C Mei J Li M Wu Y Xiang X. Lactate to albumin ratio as a prognostic marker for all-cause mortality in patients with venous thromboembolism: a retrospective cohort study. *Front Cardiovasc Med.* (2025) 12:1609295. 10.3389/fcvm.2025.1609295 41112225 PMC12528031

[20] Wang Y Feng Y Yang X Mao H. Prognostic role of elevated lactate in acute pulmonary embolism: a systematic review and meta-analysis. *Phlebology.* (2022) 37:338–47. 10.1177/02683555221081818 35282737

[21] Za̧bczyk M Natorska J Janion-Sadowska A Malinowski KP Janion M Undas A. Elevated lactate levels in acute pulmonary embolism are associated with prothrombotic fibrin clot properties: contribution of NETs formation. *J Clin Med.* (2020) 9:953. 10.3390/jcm9040953 32235490 PMC7231195

[22] Flora GD Nayak MK Ghatge M Kumskova M Patel RB Chauhan AK. Mitochondrial pyruvate dehydrogenase kinases contribute to platelet function and thrombosis in mice by regulating aerobic glycolysis. *Blood Adv.* (2023) 7:2347–59. 10.1182/bloodadvances.2023010100 36971790 PMC10230171

[23] Zhu L Dong H Li L Liu X. The mechanisms of sepsis induced coagulation dysfunction and its treatment. *J Inflamm Res.* (2025) 18:1479–95. 10.2147/JIR.S504184 39925935 PMC11804232

[24] Eckart A Struja T Kutz A Baumgartner A Baumgartner T Zurfluh S et al. Relationship of nutritional status, inflammation, and serum albumin levels during acute illness: a prospective study. *Am J Med.* (2020) 133:713–22.e7. 10.1016/j.amjmed.2019.10.031 31751531

[25] Arques S. Human serum albumin in cardiovascular diseases. *Eur J Intern Med.* (2018) 52:8–12. 10.1016/j.ejim.2018.04.014 29680174

[26] Soeters PB Wolfe RR Shenkin A. Hypoalbuminemia: pathogenesis and clinical significance. *JPEN J Parenter Enteral Nutr.* (2019) 43:181–93. 10.1002/jpen.1451 30288759 PMC7379941

[27] Hoskin S Chow V Kritharides L Ng ACC. Incidence and impact of hypoalbuminaemia on outcomes following acute pulmonary embolism. *Heart Lung Circ.* (2020) 29:280–7. 10.1016/j.hlc.2019.01.007 30975572

[28] Gök G Karadað M Çinar T Nurkalem Z Duman D. In-hospital and short-term predictors of mortality in patients with intermediate-high risk pulmonary embolism. *J Cardiovasc Thorac Res.* (2020) 12:321–7. 10.34172/jcvtr.2020.51 33510882 PMC7828758

[29] Qiu J Hao Y Huang S Wang T He X Wang W et al. Serum albumin for short-term poor prognosis in patients with acute pulmonary embolism: a clinical study based on a database. *Angiology.* (2025) 76:458–65. 10.1177/00033197241226881 38193449

[30] Suzuki Y Aoki Y Shimizu M Nakajima M Imai R Okada Y et al. Predictive accuracy of lactate albumin ratio for mortality in intensive care units: a nationwide cohort study. *BMJ Open.* (2024) 14:e088926. 10.1136/bmjopen-2024-088926 39806598 PMC11667448

[31] Ebner M Pagel CF Sentler C Harjola VP Bueno H Lerchbaumer MH et al. Venous lactate improves the prediction of in-hospital adverse outcomes in normotensive pulmonary embolism. *Eur J Intern Med.* (2021) 86:25–31. 10.1016/j.ejim.2021.01.021 33558162

[32] Vanni S Jiménez D Nazerian P Morello F Parisi M Daghini E et al. Short-term clinical outcome of normotensive patients with acute PE and high plasma lactate. *Thorax.* (2015) 70:333–8. 10.1136/thoraxjnl-2014-206300 25661114

