# Supplementary material for: Developing and validating a lactate-to-albumin ratio-enhanced score for mortality prediction in ICU patients with acute pulmonary embolism: a multi-cohort study
Source: Front Med (Lausanne). 2026 Jun 15;13:1819790. doi: 10.3389/fmed.2026.1819790 (PMC13311097; doi:10.3389/fmed.2026.1819790)
Supplement: Supplementary file 1 [file Table_1.docx]

**Table S1. Harmonized variable definitions and operationalization across all cohorts**

| **Variables** | **Operational Definition** |
| --- | --- |
| Age | Age at hospital admission (years) |
| Sex | Sex; 1 = male, 0 = female |
| Heart rate | Heart rate on admission (beats/min), first recorded within 24 h |
| SBP | Systolic blood pressure on admission (mmHg), first recorded within 24h |
| SpO2 | Peripheral oxygen saturation on admission (%), first recorded within 24h |
| Lactate | First measured value within 24 h after admission (mmol/L) |
| Albumin | First measured value within 24 h after admission (g/dL) |
| LAR | Lactate-to-albumin ratio: calculated as the ratio of first lactate to first albumin |
| sPESI | Simplified Pulmonary Embolism Severity Index, calculated within 24 h of ICU admission |
| GCS | Glasgow Coma Scale score， the lowest value within 24 h after admission |
| SAPS II | Simplified Acute Physiology Score II, calculated within 24 h of ICU admission |
| Mechanical ventilation | Use of mechanical ventilation; 1 = yes, 0 = no |
| Norepinephrine use | Use of norepinephrine; 1 = yes, 0 = no |
| Pharmacological thrombolysis | Receipt of pharmacological thrombolysis; 1 = yes, 0 = no |
| Diabetes mellitus | History of diabetes; 1 = yes, 0 = no |
| Congestive heart failure | History of congestive heart failure; 1 = yes, 0 = no |
| Chronic Pulmonary Disease | History of chronic cardiopulmonary disease; 1 = yes, 0 = no |
| Malignancy | History of malignant cancer; 1 = yes, 0 = no |
| AKI | Acute kidney injury during admission; 1 = yes, 0 = no |
|  |  |

All variables were identically defined and operationalized across the MIMIC‑IV, eICU‑CRD, and DLMU cohorts. Vital signs, laboratory parameters, and severity scores were all assessed within the first 24 h of ICU admission.
